# Pollution Risk Prediction for Cadmium in Soil from an Abandoned Mine Based on Random Forest Model

**DOI:** 10.3390/ijerph20065097

**Published:** 2023-03-14

**Authors:** Jie Cao, Zhaohui Guo, Yongjun Lv, Man Xu, Chiyue Huang, Huizhi Liang

**Affiliations:** 1 School of Metallurgy and Environment, Central South University, Changsha 410083, China; 2 Linxiang Station of Yueyang Ecology and Environment Monitoring Center, Linxiang 414300, China

**Keywords:** abandoned lead/zinc mine, random forest model, toxic metal(loid)s, cadmium, potential pollution risks

## Abstract

It is highly uncertain as to the potential risk of toxic metal(loid)s in abandoned mine soil. In this study, random forest was used to predict the risk of cadmium pollution in the soils of an abandoned lead/zinc mine. The results showed that the random forest model is stable and precise for the pollution risk prediction of toxic metal(loid)s. The mean of Cd, Cu, Tl, Zn, and Pb was 6.02, 1.30, 1.18, 2.03, and 2.08 times higher than the soil background values of China, respectively, and their coefficients of variation were above 30%. As a case study, cadmium in the mine soil had “slope” hazard characteristics while the ore sorting area was the major source area of cadmium. The theoretical values of the random forest model are similar to the practical values for the ore sorting area, metallogenic belt, riparian zone, smelting area, hazardous waste landfill, and mining area. The potential risk of soil Cd in the ore sorting area, metallogenic belt, and riparian zone are extremely high. The tendency of pollution risk migrates significantly both from the ore sorting area to the smelting area and the mining area, and to the hazardous waste landfill. The correlation of soil pollution risk is significant between the mining area, the smelting area, and the riparian zone. The results suggested that the random forest model can effectively evaluate and predict the potential risk of the spatial heterogeneity of toxic metal(loid)s in abandoned mine soils.

## 1. Introduction

Metal(loid) pollution exists in a variety of environmental mediums such as soil, water, gas, etc. [1,2]. Public poisoning due to metal pollution in the soil has been occurring worldwide [3]. The prevention of metal pollution in the soil is one of the most difficult problems to solve, which is related to the fact that metal(loid) pollution in the soil is difficult to detect and can accumulate over time [4,5]. The high accumulation of metal(loid)s within the soil will result in soil pollution that is highly regional in nature [6]. Soil pollution from mines is a typical case. Metal(loid) pollution from mines is caused by functional activities such as mining, mineral processing, smelting, etc. The areas that undertake these activities are called functional areas [7,8]. The functional area of the mine, as the source of various pollution, is an important indicator of pollution prevention. The level of pollution risk of metal(loid)s within functional areas is inconsistent, and the level of pollution risk of metal(loid)s depends on the functional behavior of different functional areas, which will lead to the spatial heterogeneity of functional areas [9,10]. In order to prevent the harmful effects of metal pollution in mine soils on public health, it makes sense to carry out risk prediction work, which is essential for the risk identification of metal(loid) pollution sources in mines and for obtaining information on the characteristics of metal(loid) pollution sources.

The phenomenon of cross-pollution between functional areas indicates that there is a major source of pollution risk in the functional areas, which requires an urgent response [11,12]. The accurate identification of pollution risk sources by classical statistical methods means using traditional full-scale sampling [13,14]. However, the disadvantage of the traditional method is the long time it takes to complete and the high expense, which is not an optimal choice for mining companies. The spatial heterogeneity of the pollution distribution of metals in soils from mines usually leads to a lot of complex data that are often difficult to interpret via traditional monitoring methods, such that metal(loid)s data from functional areas are difficult to analyze accurately and are insufficient to reveal the potential pollution risk of metal pollutants in complex environmental conditions (mines) [15]. The methods for pollution prediction, such as the human health risk assessment model [16], UNMIX model [17], or geographic information system model [18], have been established. However, compared with the above methods, random forest (RF) is considered as one of the more effective methods to provide spatial assessment and prediction, which has the advantages of requiring a small sample size, not being affected by a complex environment, and allowing researchers to dig deeper into the underlying data [19]. Wang et al. (2023) [20] applied a random forest model and land use regression model to compare the results of concentration data of six metals (Pb, Cd, Cr, As, Hg, and Zn) in agricultural soils for their advantages and disadvantages, and the validation results proved that the use of RF model is more suitable for the prediction of metal contents in agricultural soils. Azizi et al. (2023) [21] predicted the spatial distribution of some metals (Ni, Fe, Cu, and Mn) in western Iran using environmental covariates and random forest. The results demonstrate that random forest can use easily available environmental data to predict the large-scale areas under study, which is essential for decision-making on the sustainable management of environmental problems. However, to our knowledge, there are few studies that apply random forest to complex areas such as mines. Therefore, based on the above study, a proposal based on random forest for the prediction of the pollution risk of metal(loid)s in mine soils can be further proposed.

Then, for complex soil pollution, such as mine soil pollution, the RF model will be the key to obtain comprehensive information on the characteristics of toxic metal(loid) pollution sources. Therefore, the study aims (1) to evaluate the feasibility of the random forest model for the identification of the potential sources and risk characteristics of toxic metal(loid)s in soil from an abandoned lead/zinc mine, and (2) using cadmium as a case, extrapolate the potential pollution risk of various toxic metal(loid)s in the mine soil.

## 2. Materials and Methods

### 2.1. Study Area

**Figure 1:**
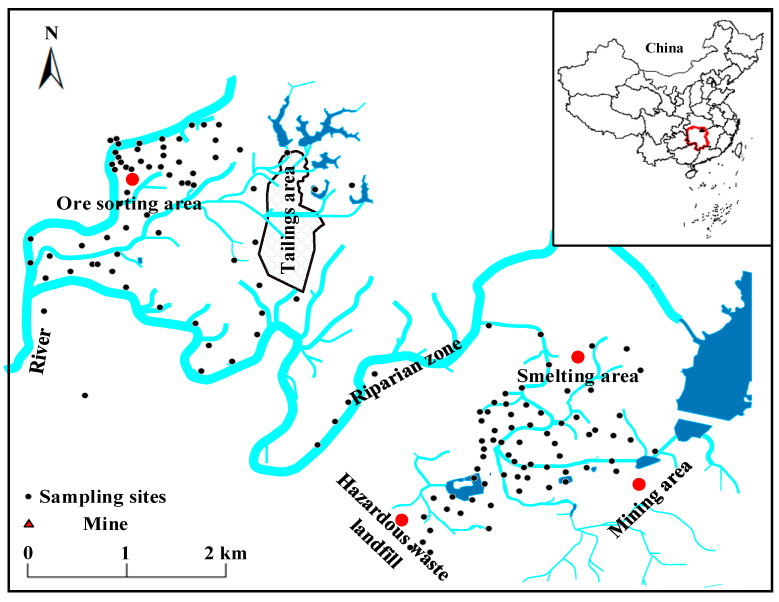
Study area and sampling sites.

The abandoned lead/zinc mine is located in central southern China, with a longitude of 113°18′ and latitude of 29°24′, and is characterized by red soil formed from quaternary laterite, slate, and shale. Due to the long-term direct discharge of industrial wastewater and the disordered stacking of waste slag, the historical legacy of the lead/zinc mine sites is one of serious pollution. There is a river running through the whole area of the mine. The flow of the river is mainly influenced by the amount of rainfall, switching to flood or dry periods with the change in seasons, and the river is the main surface runoff. Under the action of long-term water flow migration, a large number of toxic metal(loid)s are deposited in the soil and the riverbed in the vicinity of lead/zinc mine. There are seven functional areas in the mine. The ore sorting area is the area where physical and chemical measures are applied to the ore to obtain the needed ingredients for smelting or other industries. The riparian zone is the area on either side of the river–land interface until the influence of the river disappears. The hazardous waste landfill is the storage area for solid waste and industrial waste. The mining area is an area engaged in ore mining. The smelting area is the industrial area where ore calcination and refining are carried out. The tailings area is the area where the tailings or other industrial wastes after ore sorting are deposited. The metallogenic belt is a geological unit of mineral resources with potential for mineralization (Figure 1).

### 2.2. Sampling and Analysis

Soil sampling in the abandoned lead/zinc mine was designed according to the strategy of combining points, lines, and surfaces along with the river, and the screening results of pollution identification. The soil sampling was carried out at sites at intervals of about 300 m on the side flowing through the lead/zinc mine, which had to be arranged according to the direction of the mine hole and slope, combined with the flow direction of surface runoff in the mine, and two control sites had to be set in each local area of blank control. All of soil samples were collected via mechanical drilling or excavation with stainless steel shovels. A total of 147 soil samples were collected and data for ten metal(loid)s (As, Cd, Cr, Cu, Hg, Mn, Sb, Tl, Pb, and Zn) were determined, with 147 pieces of data for individual metal(loid)s and a total of 1470 pieces of data obtained.

Each of the 147 soil samples was placed indoors for air-drying for 7 days. The samples were ground and then filtered through a 2 mm sieve and prepared for use. Soil samples were digested with mixed acid (HCl-HNO_3_-HClO_4_). The procedure was as follows: 1.0 g of soil sample was mixed with 5.0 mL of concentrated nitric acid (HNO_3_), 3.0 mL of concentrated hydrochloric acid (HCl), and 2.0 mL of concentrated perchloric acid (HClO_4_), and digested using microwave at 160 °C for 2 h.

Inductively coupled plasma mass spectrometry (ICP, iCAP 7600, Thermo Scientific, 81 Wyman Street, Waltham, MA, USA) was used to determine the concentrations of As, Cd, Cr, Cu, Hg, Mn, Sb, Tl, Pb, and Zn in the digested solution. The experiments were carried out on the reagent blank group and repeated soil samples to check the accuracy of the experimental method and data. The recovery rate was 100 ± 10%. The analytical method was tested using the national first-class soil standard material (HJ 25.1-2019) of the People’s Republic of China. The background values (As:13.6, Cd:0.081, Cr:71.4, Cu:25.4, Hg:0.087, Sb:1.58, Tl:0.61, Pb:27.3, and Zn:88.6) for soil metal(loid)s were based on the CNEMC (China National Environmental Monitoring Center), Beijing, China, 1990 [22].

### 2.3. Modeling of Random Forest

A random forest is a non-parametric model that iteratively classifies or regresses data to find the best split point, generates N decision trees, and finally uses a voting mechanism in the forest to determine the output. Random forest are characterized by randomly selected features and samples, allowing each tree in the forest to have similarities and differences. The bootstrap method is used to randomly draw k new sets of self-help samples with put-back from the original training dataset, and from this, k classification regression trees are constructed, and each undrawn sample forms K out-of-bag data (OOB_1_, OOB_2_,…, OOB_k_). Given n features, n features are randomly selected at each tree node. The feature with the highest classification power is chosen for node splitting by calculating the amount of information contained in each feature. The impact of each feature on the model’s accuracy rate is directly measured during feature selection. The prediction accuracy OA_1_, OA_2_,…, OA_K_ for k out-of-bag datasets is obtained by inputting each out-of-bag dataset into the corresponding decision tree. For the assessment index f_i_, the index values under this assessment index are randomly replaced in all out-of-bag datasets, while the other index values remain unchanged, resulting in the new out-of-bag datasets OOB_1_^i^, OOB_2_^i^,…, OOB_k_^i^. After random replacement of the evaluation indicator f_i_, OA_1_^i^, OA_2_^i^,…, OA_K_^i^, they are input into the decision tree corresponding to step to determine the prediction accuracy of the out-of-bag dataset. 

The random forest model is based on the background of big data information and is not affected by complex environments. In this work, 147 soil samples were randomly divided into modeling set and validation set, this step was conducted to build and validate the model in the same batch of data to improve its validity. A total of 130 samples were used as the modeling set to build the model and 17 samples were used as the validation set to illustrate the feasibility of the model after the validation of the data samples. With toxic metal(loid) content in the soil as the dependent variable, the model was established using random forest, and the prediction was made according to the validation set. By referencing the sklearn library, it was possible to construct classifier objects, training sample sets, predicted values, and complete evaluation, in four steps. The stability and accuracy of the model were analyzed by the coefficient of determination (R^2^). When R^2^ was closer to one, the fitting effect of the regression equation was better and the model was more stable. 

## 3. Results and Discussion

### 3.1. Pollution Characteristics of Toxic Metal(loid)s in the Mine Soil

The difference between the minimum and maximum values of 10 metal(loid)s (As, Cd, Cr, Cu, Hg, Mn, Sb, Tl, Zn, and Pb) in the soil of the abandoned lead/zinc mine is too high and thus affects the mean values (Table 1). The median values for Cd, Cu, Tl, Zn, and Pb were 6.02, 1.30, 1.18, 2.03, and 2.08 times higher than the background values, respectively, indicating that five types of toxic metal(loid) (Cd, Cu, Tl, Zn, and Pb) pollution exist in the soil [23]. The coefficient of variation of these data provides a better indication of the degree of dispersion compared to standard deviation, and the coefficients of variation for the 10 metals (As, Cd, Cr, Cu, Hg, Mn, Sb, Tl, Pb, and Zn) were above 15%, with Cd being the highest value, indicating a higher level of risk for toxic metal(loid)s in the soil. According to the above analysis, Cd is considered to be the main contaminant at risk in abandoned mine [24].

**Figure 2:**
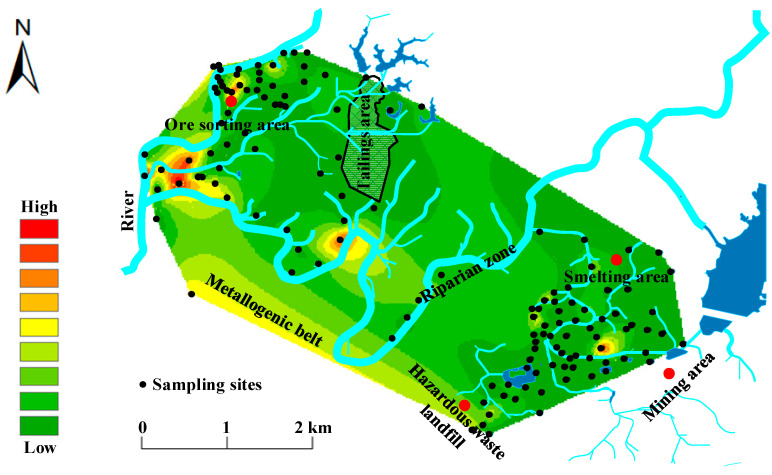
Spatial distribution of cadmium pollution in the soil from an abandoned lead/zinc mine.

The variability of cadmium pollution distribution in the lead/zinc mine soil is great, with severe pollution in the ore sorting area and metallogenic belt. The degree of soil pollution shows a decreasing trend centered on the ore sorting area and metallogenic belt (Figure 2), and there is some correlation between the sources of pollution, which is related to human activities such as early and unreasonable exploitation of mineral resources and the random stacking of tailings [25,26]. Moreover, there may be some correlation between the ore sorting area, metallogenic belt, and riparian zone, according to the division of the water system; there is a high pollution area block in the upstream ore sorting area, while the metallogenic belt and riparian zone are located downstream, and there is an obvious decreasing trend of the pollution block from the ore sorting area to the metallogenic belt and riparian zone. Therefore, based on the flow direction of the water system and the gradually decreasing spatial content, it is inferred that the ore sorting area and the metallogenic belt and riparian zone may have mutual pollution [27]. This can be attributed to the activities of mineral processing, smelting, and solid disposal, and the chaos of wastewater treatment and drainage that severely pollutes the surrounding soil. The area heavily polluted by cadmium is mainly situated in the ore sorting area, where cadmium in the soil is the source of pollution, and the pollution from the ore sorting area to the mining area shows a gradually decreasing trend, which is related to unreasonable exploitation of mineral resources at an early stage and randomly stacking residues [28]. Secondly, cadmium pollution from hazardous waste landfill and smelting areas has a more serious impact on the soil, as well as in mining areas under the movement of surface water and groundwater.

### 3.2. Validation of Random Forest Model

**Figure 3:**
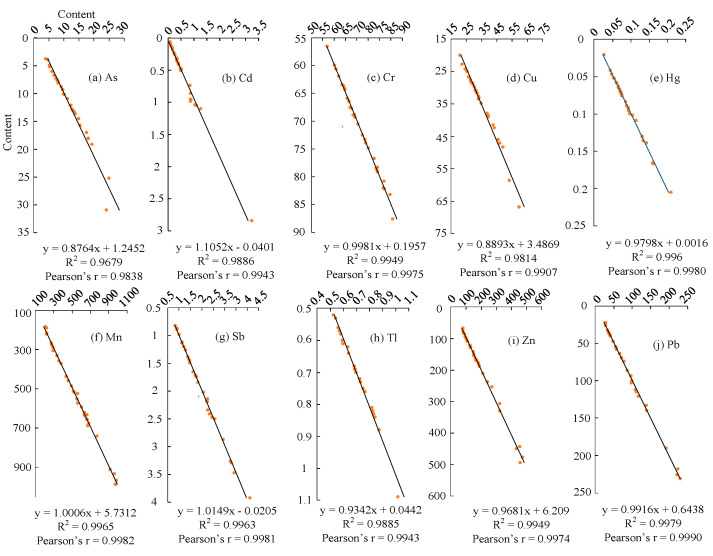
Feasibility verification of random forest model.

In this study, based on the content of 10 elements in 130 groups, a total of 1300 pieces of data was substituted into the random forest model, and the feature values were selected according to a quarter of the total number of sample feature variables. Through the feasibility check, R^2^ of the elements (As, Cd, Cr, Cu, Hg, Mn, Sb, Tl, Pb, and Zn) were all found to exceed 0.95, the theoretical value of the model was very consistent with a practical value (Pearson’s r > 0.98), and the feasibility of constructing the model was passed (Figure 3).

Unconstructed groups of 17 pieces of data were selected as the validation of the prediction model, and groups of 17 pieces of data were substituted into the random forest model to obtain the theoretical values of cadmium and compare them with the practical values. The R^2^ was as high as 0.81, and the pattern of similarity was essentially the same, which demonstrated the superiority of the random forest model in the cadmium prediction task. The mean error was less than 1%, indicating a high degree of coherence between the two data sets [29], and the median error was below 10% (Table 2), indicating a strong similarity between the two data sets. Results from all three indicators show that the random forest model has an obvious recognition capability and high accuracy for predicting toxic metal(loid)s [30,31]. When data cannot normally be obtained due to environmental and geological factors in the regional soil, the random forest model can still achieve high predictive precision [32,33]. The results show that the random forest model is effective at predicting soil cadmium levels, confirming the science and the advance of the random forest prediction model.

### 3.3. Spatial Risk Prediction of Cadmium Pollution in the Mine Soil

Pollution risk assessment in soils is an important tool for environmental prevention and control [34]. According to the theoretical values of cadmium, the high-risk area of the mine for cadmium is the ore sorting area, which shows abnormally high values, and the distribution of the surrounding soil shows a progressive decline in risk, which indicates that production behavior has impacted the accumulation of cadmium in the mine soil, and steps should be taken in advance to intercept and control it to avoid a progressive increase in the environmental risk of the surrounding soil [35]. The metallogenic belt and riparian zone are adjacent to the ore sorting area and have similar tendency of pollution risk for cadmium, confirm that the ore sorting area is a significant potential soil pollution source in the metallogenic belt and riparian zone. Therefore, effective soil control measures should be taken from the sources of pollution in the ore sorting area in order to eliminate pollution caused by the migration of soil pollution through the water stream, thus reducing the level of pollution risk in the metallogenic belt and riparian zone. Furthermore, the variability of cadmium in different functional areas confirms the spatial heterogeneity of the distribution of cadmium pollution in the lead/zinc mine soil [36].

**Figure 4:**
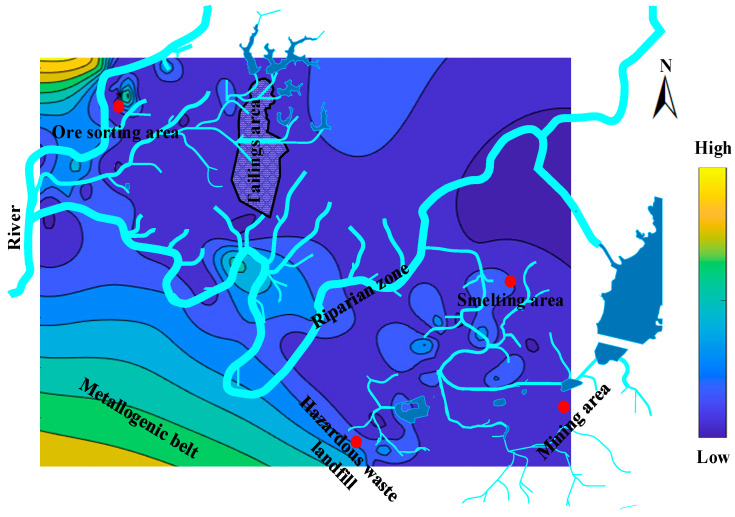
Spatial risk distribution of cadmium pollution in the soil from an abandoned lead/zinc mine.

The mine pollution hazard is characterized by a “slope” from the ore sorting area to the metallogenic belt (Figure 4). The strong correlation between the risk of soil pollution in the mining area, smelting area, and the riparian zone means that cadmium in riparian zone soils can migrate into the river and pollute the soils in mining and smelting areas. As a result, the riparian zone should be blocked to mitigate the level of soil risk in the mining area and smelting areas.

### 3.4. Validation of Spatial Pollution Risk Prediction for Cadmium

The practical values of toxic metal(loid)s in 147 sets of soil samples were used as modeling sets in a random forest model. Cadmium was used as an independent validation set (a total data of 147), and the prediction model of the cadmium content in soil and a spatial prediction model was constructed based on the modeling set. Finally, based on the measured cadmium content, the content and spatial prediction were verified [37].

**Figure 5:**
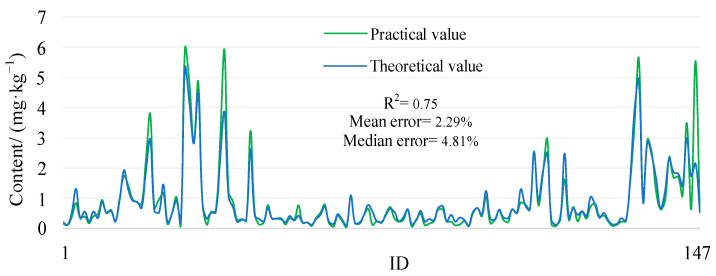
Comparison of risk values for spatial pollution of cadmium in soil from abandoned lead/zinc mine.

The optimum precision in inversion modelling was high, where the cadmium R^2^ was 0.75, and the pattern of adjustment was essentially the same. Of these, both groups of data with mean and median error exhibited a high degree of coincidence, and the errors in the two data sets were less than 5% (Figure 5), which indicates that the similarity between the two data sets was extremely high [38,39]. It has been demonstrated that the random forest model has good stability and high inversion accuracy in the cadmium forecasting task. In conclusion, the random forest model has a clear recognition capability and a high precision in the prediction of cadmium from toxic metal(loid)s.

**Figure 6:**
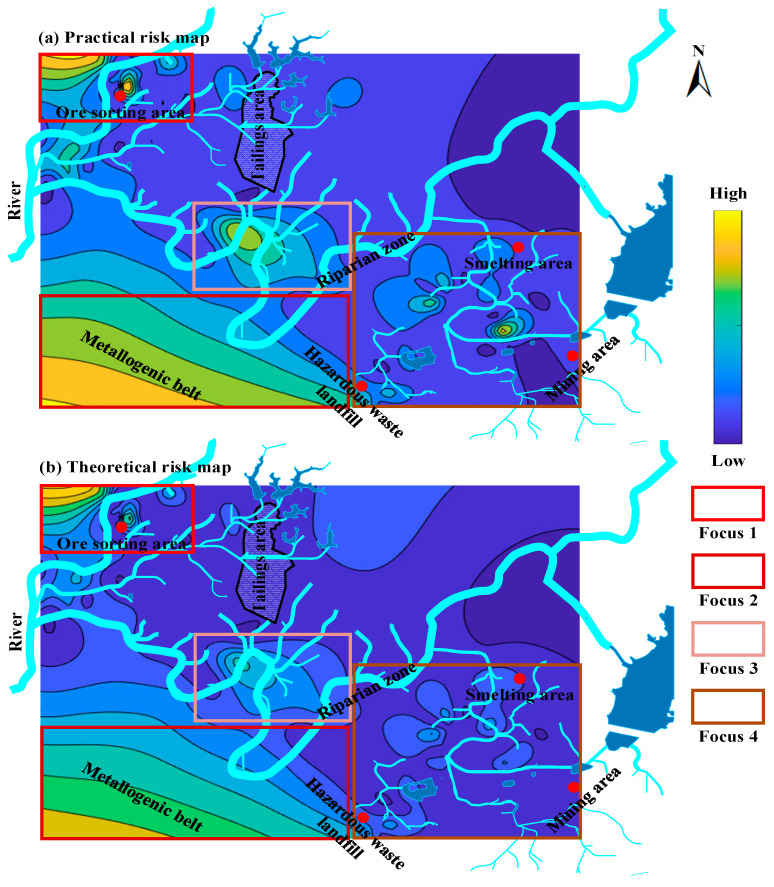
Spatial distribution of cadmium pollution risk in soil of abandoned lead/zinc mine.

The theoretical values of the spatial risk distribution of cadmium in soil are similar to the practical values. According to a cadmium spatial pollution risk in soil comparison between Figure 6a,b, the risks for the ore sorting area (Focus 1), metallogenic belt (Focus 2), and riparian zone (Focus 3) are high. The risk for the smelting area, mining area, and hazardous waste landfill (Focus 4) are low. The similarity between Focus 1 to 4 is very high, and the ore sorting area is still the highest risk area among them, indicating that the functional behavior of the ore sorting area has a serious impact on the spatial distribution of cadmium in soil. The diffusion trend of cadmium pollution risk is similar and migrates obviously from the ore sorting area to the smelting area and mining area, and hazardous waste landfill, which proves that the ore sorting area has caused serious pollution to the downstream soil of the lead/zinc mine. The results suggest that the random forest model has shown great stability in cross-validation and a strong capacity for generalization and a high predictive precision in independent validation. The predictive model was very precise and stable, and the theoretical values were valid. The high-risk area of cadmium in the soil is located in the ore sorting area and should be carried out to prevent the pollution from migrating with surface water and groundwater.

In the future, soil risk prediction for complex areas such as mines can be predicted using random forest at a large scale. From an effectiveness point of view, the current results basically meet the needs, which will help the government and enterprises to carry out the identification of risk sources. Based on this work, the ore sorting area is a high-risk area for cadmium in abandoned lead/zinc mines. Therefore, the risk prediction of priority polluting metals can be conducted separately from the risk prediction of lead/zinc mines, and, finally, the high-risk areas of metals can be integrated to obtain the prevention and control areas that should be given priority.

## 4. Conclusions

(1) A random forest model can identify the unequal distribution of toxic metal(loid) data in soil, and effectively predict the spatial heterogeneity and the potential pollution risk of soil cadmium in a heavily polluted area from an abandoned mine.

(2) According to random forest model, the theoretical values of As, Cd, Cr, Cu, Hg, Mn, Sb, Tl, Pb, and Zn in the mine soil corresponded perfectly to the practical values and may be used to predict the contents of toxic metal(loid)s (As, Cd, Cr, Cu, Hg, Mn, Sb, Tl, Pb, and Zn).

(3) The random forest model is universal for the spatial prediction of toxic metal(loid) pollutants under complex environmental conditions. The ore sorting area is the source of the pollution risk for cadmium, and the level of risk shows a downward trend from the ore sorting area to the smelting area, mining area, and hazardous waste landfill.

## Figures and Tables

**Table 1:** Characteristics of the content of toxic metal(loid)s in the soil of the abandoned mine.

Metal(loid)s	Minimum	Median	Maximum	Mean	SD ^a^	CV ^b^ (%)	BV ^c^
As	1.31	10.00	37.5	11.23	6.61	58.89	13.6
Cd	0.039	0.49	5.98	0.91	1.22	133.32	0.081
Cr	29.8	70.00	220	72.37	17.47	24.13	71.4
Cu	4.9	33.00	204	40.91	28.13	68.76	25.4
Hg	0.012	0.082	0.52	0.11	0.09	79.97	0.087
Mn	148	484.00	2230	528.53	287.40	54.38	/
Sb	0.23	1.54	6.73	1.80	1.09	60.94	1.58
Tl	0.47	0.72	2.15	0.77	0.24	31.88	0.61
Zn	49.8	180.00	1320	269.69	241.52	89.56	88.6
Pb	ND ^d^	56.7	289.00	77.12	62.75	81.37	27.3

^a^ SD: Standard deviation; ^b^ CV: coefficient of variation; ^c^ BV: background value ^d^ ND: not detected, and not participating in validation.

**Table 2:** Verification of model construction.

**ID**	**1**	**2**	**3**	**4**	**5**	**6**	**7**	**8**	**9**
Practical value	1.48	3.35	5.64	0.94	2.95	2.49	1.18	0.63	0.89
Theoretical value	1.47	3.63	4.34	1.2	2.4	2.41	1.8	1	1.18
**ID**	**10**	**11**	**12**	**13**	**14**	**15**	**16**	**17**	
Practical value	2.37	1.67	1.72	1.07	3.49	0.64	5.54	0.53	
Theoretical value	2.67	1.86	1.8	1.41	2.81	1.12	4.49	1.23	

## Data Availability

The data that support the findings of this study are available on request from the corresponding author.
